# Editorial: Emerging roles of N6-methyladenosine (m6A) modifications in endocrine disorders

**DOI:** 10.3389/fendo.2023.1330202

**Published:** 2023-11-28

**Authors:** Pengli Bu, Kamyar Asadipooya

**Affiliations:** ^1^ Department of Pharmaceutical Sciences, College of Pharmacy and Health Sciences, St. John’s University, New York, NY, United States; ^2^ Department of Medicine, Division of Endocrinology, Diabetes, and Metabolism, Barnstable Brown Diabetes and Obesity Center, University of Kentucky, Lexington, KY, United States

**Keywords:** N6-methyladenosine, microRNA, ER stress, diabetes, diabetic nephropathy

Diabetic kidney disease, or diabetic nephropathy (DN), is the main cause of end-stage kidney disease in developed countries (1). DN is considered a complication at the microvascular level, and it occurs in patients with both type 1 and type 2 diabetes mellitus (DM). Sensitive and reliable methods allowing early diagnosis of DN are therefore pivotal in initiating treatment to halt the progression of DN, which, if left untreated, will lead to end-stage kidney disease. Combining the available scientific literature and the continual advancement of biomedical research with the amazing power of artificial intelligence, previous high throughput and genome-wide datasets are now a rich resource for discoveries of novel biomarkers of disease and conditions when employing machine learning algorithms for second analyses. This emerging trend has been well exemplified in the Research Topic “*Emerging Roles of N6-methyladenosine (m6A) Modifications in Endocrine Disorders*”, which contains a selection of one review and four original research articles. The review paper summarizes the connections between ER stress and DN (Yang et al.); one research article, utilizing the traditional biomarker research approach, identifies urine m6A modification level as a novel biomarker for DN (Wan et al.); and three research articles employ machine learning methods to re-examine available datasets for mechanism-based biomarker discoveries (Li et al.; Su et al.; Zhang et al.).

In the research paper by Wan et al., m6A modification levels were quantified in the urine of age- and gender-matched subjects of normal glucose tolerance, type 2 diabetes mellitus (T2DM), and DN. The authors reported a negative association between urine m6A modification levels and the clinical severity of DN. This finding could add another tool to the current armamentarium of DN diagnosis with the benefit of non-invasive sample collection. Among the many advancements in recent biomedical research, the emergence of microRNA (miRNA) is certainly a notable one. These small, non-coding RNA molecules have been shown to play crucial roles in an array of physiological and pathological conditions (2, 3). By employing multiple machine learning algorithms, Li et al. retrieved and analyzed PubMed research papers from 1993 to 2023 on the topic of miRNA and diabetes through topic modeling, biomedical term tagging, Train-test data set splitting and machine learning analysis, and pathway constructing. The authors identified 13 distinct topics of miRNA studies and discovered miR-146 as one of the critical biomarkers for diabetes prediction, as miR-146 is involved in multiple pathways and connected to genes important for diabetic inflammation and neuropathy. With a comprehensive background laid down by the review article (Yang et al.), the readers will grasp the critical importance of ER homeostasis in physiology (i.e., the normal/healthy state) and understand why ER stress is considered a critical disease trigger and an amplifying event in DN. The readers can then transition to the research paper by Su et al. to appreciate the significance of the discovery made via bioinformatics and machine learning of the 5 ER stress-related signature genes as novel biomarkers for DN. Last but not least is the research paper connecting m6A modification to obesity and the pathogenesis of ossification of ligamentum flavum (OLF). Zhang et al. utilize integrative bioinformatic algorithms to identify 8 obesity-related differentially expressed genes (ORDEGs), 14 differential OLF-related infiltrating immune cells (OIICs), 6 m6A modifiers, and 4 5mC regulators in OLF. This study deciphers inflammatory signature genes in obesity that can potentially be utilized as biomarkers and/or therapeutic targets of obesity-related OLF and Sheds light on the association between 5mC/m6A modification and SOCS3 expression and immune infiltration.

## Author contributions

PB: Conceptualization, Supervision, Writing – original draft, Writing – review & editing. KA: Conceptualization, Writing – review & editing.
